# DFT, hirshfeld and molecular docking studies of a hybrid compound - 2,4-Diamino-6-methyl-1,3,5-triazin-1-ium hydrogen oxalate as a promising anti -breast cancer agent

**DOI:** 10.1016/j.heliyon.2022.e10355

**Published:** 2022-08-24

**Authors:** K. Ayisha Begam, N. Kanagathara, M.K. Marchewka, An-Ya Lo

**Affiliations:** a Department of Physics, Saveetha Engineering College, Chennai, 602 105, India; b Department of Physics, Saveetha School of Engineering, Saveetha Institute of Medical and Technical Sciences, Thandalam, Chennai, 602 105, India; c Institute of Low Temperature and Structure Research, Polish Academy of Sciences, 50-950 Wrocław, 2, P.O. Box 937, Poland; d Department of Chemical and Materials Engineering, National Chin-Yi University of Technology, Taichung, 411030, Taiwan

**Keywords:** DFT—B3LYP, Natural bond orbital (NBO), HOMO-LUMO, NLO, Hirshfeld

## Abstract

The six-membered heterocyclic ring - 1,3,5-triazine and its derivatives have garnered a lot of attention because they're good bioactive herbicides, cancer agents, and other things. One such triazine derivative, 2,4-diamino-6-methyl-1,3,5-triazin-1-ium hydrogen oxalate (DMTHO), was produced in this work, and the structure was optimised using density functional theory's B3LYP functional and the basis set 6–31++G (d,p). Additionally, the chemical underwent in-depth research using molecular docking analysis, Hirshfeld, and density functional theory. The electron densities distribution in the atoms is provided by natural orbital analysis, which also characterises the chemical bonding and reaction behaviour of the compound. The calculated HOMO and LUMO energies indicate that charge transfer occurs inside the molecule. Chemical reactivity traits including HOMO-LUMO energy gaps, softness, total energy, chemical hardness, electronic chemical potential, and electrophilicity of bioactive substances have all been subjected to analytical investigation. Total dipole moment (μ) and first-order hyperpolarizability (β) measurements for the investigated chemical indicate that DMTHO may exhibit microscopic nonlinear optical (NLO) behaviour with nonzero values. A quantitative description about intermolecular interactions in the produced crystal is provided by the Hirshfeld surface analysis. Further docking studies of the compound have been performed and the results reveals that the compound inhibit the breast cancer related protein - casein kinase (CK2) – and the possibility of developing as a potential anti breast cancer lead.

## 1 Introduction

Pyridazinone as well s-triazine derivatives are a class of nitrogenous heterocyclic chemicals, have drawn a lot of attention because of its potential therapeutic and pharmacological effects as anti-tumor, anti-inflammatory, anti-fungal, antidepressant, anticonvulsant, anti-tubercular and antiviral agents [1, 2]. Triazine derivatives are effective antiviral, antibacterial, and antiparasitic medications [3]. The 1,3,5-triazine moiety continues to draw a lot of interest as a building block for new substances and materials with a variety of beneficial features, from biological activity to liquid crystals. Computational studies for π -π interactions of several electron-deficient systems, including 1,3,5-triazines and tetrazines, were conducted. The 2,4,6-triamino-1,3,5-triazines' significant affinity for graphite was demonstrated by DFT simulations, and the driving force for adsorption is an attractive contact between the amino groups and the underlying surface [4]. The triazines are among of the earliest organic heterocycles with nitrogen that are known to science. Although 1,3,5-triazines are best recognised as anticancer medications, they are also present in a number of other areas of medicinal chemistry [5]. Earlier reports reveals that triazine and its derivatives include complementary arrays of hydrogen-bonding sites, which allows them to create non-covalent supramolecular structures with numerous hydrogen bonds [6, 7]. 2,4-diamino 6-methyl 1,3,5-triazine is one such derivative with commercial, chemical, and pharmaceutical uses. Research on triazine salts with various organic and inorganic acids has been done in several research [8, 9, 10, 11]. In continuation of research on such series, one such s-triazine derivative 2,4-diamino 6-methyl 1,3,5-triazinium hydrogen oxalate is taken as the subject of investigation.

Oxalic acid, a member of the dicarboxylic acid family, is suitable for research in photonics, which requires the immediate fusion of two carboxyl clusters. Oxalate derivatives are used in both industrial and medical settings and act as bridge components for molecular assemblies and polymer complexes [12]. Numerous researches have reported on the generation and characterisation of pure oxalic acid as well as oxalic acid doped with amino acids and other bases for their nonlinear optical applications [13, 14, 15, 16]. Begam et al. published their findings on the structural and vibrational analysis of trifluoroacetic acid and levulinic acid with 2,4-diamino 6-methyl 1,3,5-triazine [16, 17]. The research on 2,4-diamino 6-methyl 1,3,5-triazine with oxalic acid uses for this work. The crystal structure of 2,4-diamino 6-methyl 1,3,5-triazinium hydrogen oxalate (DMTHO) was described by Narimani and Yamin [18]. The majority of materials with NLO properties have healthy biological activities. Interesting structural and conformational characteristics, as well as a noteworthy biological activity, are displayed by s-triazine. Recently, Kanagathara et al. published on the structure and vibrational properties of the chosen chemical, DMTHO [19]. Regarding other investigations like NBO, HOMO-LUMO, NLO, Hirshfeld, and molecular docking, there were no reports available. Thus, using DFT/B3LYP-6-311++G (d,p), we provide the calculations of the frontier molecular orbital, natural bonding orbital, and first-order hyperpolarizability for DMTHO here. Hirshfeld surface analysis was utilised to determine the intermolecular interaction between the compounds. In order to ascertain the biological activity of the chosen chemical, molecular docking experiments on cancer disease proteins are conducted.

## 2 Materials and computational methods

DMTHO crystals were made using oxalic acid and 2,4-diamino-6-methyl-1,3,5-triazine of analytical grade in a 1:1 ratio. Both solutes were dissolved in water, which served as their solvent, and their mixture produced a transparent, homogenous solution. After that, it was left to evaporate at ambient temperature. After one month, well-formed DMTHO crystals were harvested.

Using the 6–311++G (d,p) basis set and the Gaussian 09 programme, quantum mechanical calculations were carried out to determine the structure and vibrational wavenumbers of the generated crystals [20]. Natural bond orbital (NBO) energies and HOMO-LUMO orbital energies were investigated, and the Gauss View 4.1 programme was used to obtain a visual depiction of the aforementioned features [21, 22]. The oscillator strengths, HOMO-LUMO energies, and permitted excitations were all included in the electronic absorption spectrum. Density functional theory was used to compute the dipole moment, mean polarizability, and first static hyperpolarizability based on the finite field approach [23]. Crystal Explorer 3.0 was used to create 2-D fingerprint plots and Hirshfeld surface analysis, two useful methods for defining a crystal structure's surface properties [24]. The molecular docking studies were the preferred method of auto dock vina software [25]. For the study of the docking outcomes, BIOVIA Discovery Studio Visualization software [26] was utilised.

## 3 Results and discussion

### 3.1 Single crystal XRD (SCXRD) studies

The harvested DMTHO crystal was subjected to single-crystal XRD analysis using a KUMA 4 diffractometer. SCXRD studies reveals the presence of DMTHO crystals in the triclinic system with space group P-1, and the unit cell parameters were, a = 5.6208 (12) Å, b = 7.9828 (17) Å, c = 10.857 (2) Å, α = 76.846 (4)°, β = 75.882 (4)°, γ = 75.954 (4)°, and V = 450.92 (17) Å^3^. These values are well supported by the literature [18, 19].

### 3.2 Natural bond orbital (NBO) studies

NBO analysis is a useful technique for examining the interactions between molecules' intra- and intermolecular bonds. The lone pair Lewis-type NBO and the Rydberg non-Lewis bonding NBO are examples of NBOs that convey information about the interaction between full and empty antibodies. Additionally, it offers a convincing defence for investigating the conjugative interaction or charge transfer in molecular systems [27]. Electron donor and acceptor interactions grow as the stabilisation energy “E (2)” rises. The delocalization of electron density between occupied Lewis-type (bond or lone pair) NBO orbitals and formally empty (antibond or Rydgberg) non-Lewis NBO orbitals is consistent with a stabilised donor-acceptor interaction [28]. Second-order perturbation Fock matrix theory was used to compute the donor-acceptor interactions of the DMTHO molecule, and the results are shown in Table 1. The delocalization of electrons from filled Lewis-type NBO to non-Lewis-type NBO is revealed by the hyperconjugation phenomenon.

**Table 1:** Second-order perturbation theory analysis of the Fock matrix in NBO basis calculated at B3LYP/6–311++G (d,p) level.

	Acceptor (j)	E^(2)^ (KJmol^−1^)	E(j)-E(i) (a.u)	F (i.j) (a.u)
**Within Unit 1**				
BD (2)C1–N10	BD∗(2)C2–N9	33.96	0.34	0.105
BD (2)C2–N9	BD∗(2)C3–N11	39.30	0.31	0.097
BD (2)C3–N11	BD∗(2)C1–N10	47.34	0.27	0.110
LP (1)N11	BD∗(2)C3–N9	12.26	0.83	0.091
LP (1)N12	BD∗(2)C1–N10	97.60	0.20	0.132
BD∗(2)C1–N10	BD∗(2)C2–N9	42.45	0.05	0.062
LP (1)N9	BD∗(1)C2 –N10	12.19	0.84	0.091
LP (1)N13	BD∗(2)C3–N11	64.02	0.26	0.119
LP (1)N11	BD∗(1)C3–N9	12.26	0.83	0.091
BD (2)C3–N11	BD∗(2)C2–N 9	157.08	0.02	0.071
**From Unit 2 to Unit 1**				
LP (1)O22	BD∗(1) H4 – N10	11.00	0.92	0.093
LP (2)O22	BD∗(1) H4 – N10	66.36	0.68	0.192
LP (1)O23	BD∗(1)N12–H16	7.66	1.07	0.081
LP (2)O23	BD∗(1) N12 – H16	23.23	0.69	0.116
**Within Unit 2**				
LP (2)O20	BD∗(1)C18–O19	20.26	0.62	0.101
LP (2)O20	BD∗(1)C19–O21	32.08	0.62	0.128
LP (1)O22	BD∗(1)C18–O23	8.30	1.17	0.088
LP (2)O21	BD∗(1)C19–O20	43.46	0.36	0.112
LP (2)O22	BD∗(2)C18–O19	13.49	0.72	0.090
LP (3)O22	BD∗(1)C18-023	87.02	0.29	0.142
LP (2)O23	BD∗(1)C18–C19	18.91	0.64	0.100
LP (2)O23	BD∗(1)C18–O22	13.78	0.82	0.098

The stabilisation energy is E (2) and is given in Eq. (1) as follows for each donor (i) and acceptor (j).

(1) $$\text{E}2\text{ ​}=\text{ ​}\Delta \text{Eij}=\text{qi}\frac{F\left(i,j\right)2}{Ei-Ej}$$

where qi is the donor orbital's occupancy, Ei and Ej are the orbitals' respective energies, and F (i,j) is a component of the NBO Fock matrix.

**Figure 1:**
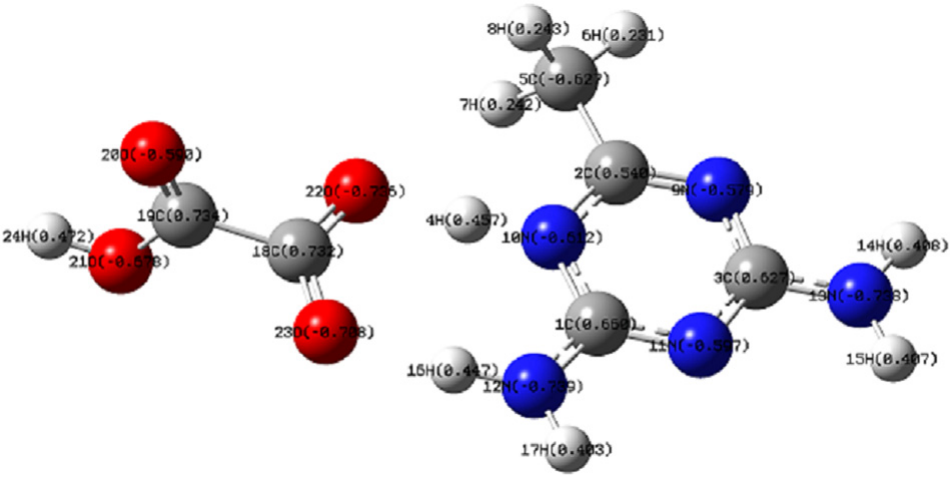
NBO charges of DMTHO.

As shown in Figure 1, all the carbon atoms possess positive charges except 5C, which possess a negative charge of 0.627 e. The carbon atom 5C forms a methyl group with three hydrogen atoms 6H, 7H, and 8H (approximately 0.243 e), and it is attached to another carbon atom 2C (0.540 e). Similarly, the hydrogen atoms 14H, 15H, and 17H attached to nitrogen in the triazine rings have a positive charge of approximately 0.40 e. The protonated 4H and 16H atoms connected to the 10N atom of negative charge of -0.612 e and 12N of charge -0.739, respectively, in the triazine ring have positive charges of approximately 0.45 e. Additionally, the oxalate group's oxygen atoms all carry negative charges. The oxygen atoms 22O and 23O of charge -0.7 e are attached to the carbon atom 18C of charge 0.732 e. The oxygen atom 21O, which is attached to the hydrogen atom 24H with charge 0.472 e, has a negative charge of 0.678 e. The oxygen atoms 21O and 20O (−0.590) are attached to the carbon atom 19C of charge 0.734 e. The strongest intra molecular interaction energy within the DMT cation was 157.08 kJ mol^−1^ which is assigned to the transition from π(C3–N11) to π∗(C2–N9). This supports electrostatic potential studies' conclusion that the electron cloud can build up around the carbon atom [29, 30]. The next most substantial stabilization energies were 97.60 kJ mol^−1^ and 87.02 kJ mol^−1^ due to the intramolecular hyperconjugative interaction of a lone pair of nitrogen atom N12 with π∗(C1–N10) and a lone pair of 22O with the anti-bonding π∗(C18–O23), respectively. The lone pair of nitrogen atom (N13) with π∗ (C3–N11) has an intramolecular stabilization energy transition value of 64.02 kJ mol^-1^ in the DMT unit. The intermolecular stabilization energy of 66.36 kJ mol^−1^ is assigned to the lone pair interaction of oxygen O22 with the anti-bonding σ∗(H4–N10). The next highest intermolecular stabilization transition was from the interaction of lone pair of oxygen atom O23 with σ∗(N12–H16) with an energy value of 23.23 kJ mol^-1^. These results revealed the strong inter- and intramolecular stabilization energies of the studied compound. Table 1 shows the influence of available strong stabilization energies and inter/intra molecular interactions in the crystal packing of the chosen material. According to earlier research cited by Mahmood Asif et al., intramolecular hydrogen bonding causes an increase in antioxidant activity [31].

### 3.3 Frontier molecular orbitals studies

**Figure 2:**
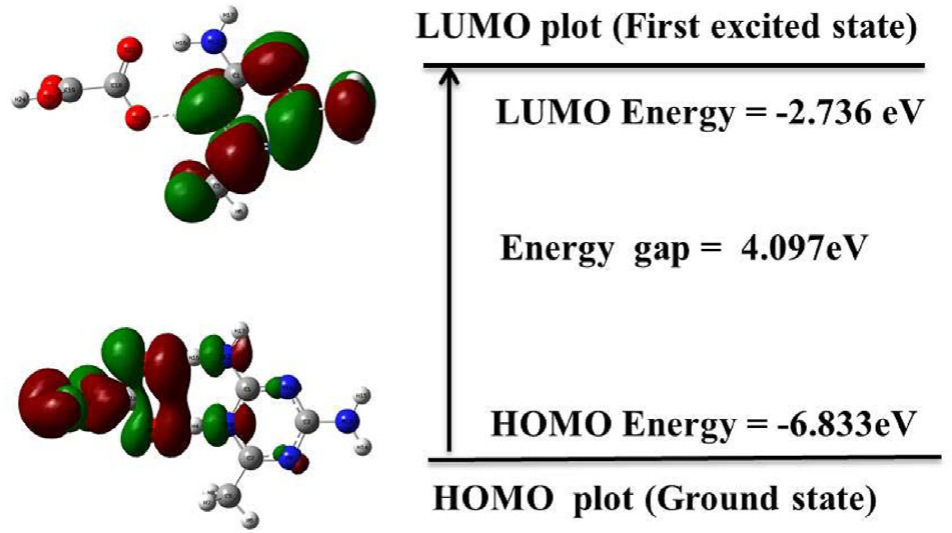
HOMO-LUMO plot of DMTHO.

More information on the material's electrical and optical properties is provided by the frontier molecular orbital, which also explains the various types of reactions that can take place in conjugated systems [32]. The highest occupied molecular orbital (HOMO) represents the capacity to give an electron, whereas the lowest unoccupied molecular orbital (LUMO) represents the propensity to gain an electron (nucleophile). Using HOMO and LUMO energy values, several global chemical reactivity descriptors of molecules, such as hardness (η), chemical potential (μ), softness (S), electronegativity (χ), and electrophilicity index (ω) were calculated [33, 34]. Figure 2 represents the HOMO and LUMO molecular surfaces.

The LUMO is localized in the DMT cation with a calculated energy of = -2.736 eV, whereas the HOMO is localized mainly in the oxalate anion with HOMO = -6.833 eV. The HOMO-LUMO energy gap was computed to be 4.097 eV. The high energy gap implies that the grown crystal is highly stable, chemically less reactive, and less polarized. The other parameters were computed and are listed in Table 2. The ionization potential is related to HOMO energy value and electron affinity is related to LUMO energy values with positive signs. Higher ionization potential and electron affinity of an element indicates higher electronegative value of the element. Usually, the electron affinity is less than the ionization potential, which implies that the molecule has a strong ability to gain electrons. The ability of the molecule to absorb electrons is measured by the global electrophilicity index, which is based on chemical potential and chemical hardness. The low electrophilicity (0.4385 eV) also supports the high stability and bioactivity of the grown material [35]. The stability index of the compounds was determined by the HOMO-LUMO energy gap, which also provides information on the wavelength at which the molecule may absorb.

**Table 2:** Calculated frontier molecular orbital analysis of the 2,4-diamino 6-methyl 1,3,5-triazine oxalate compound.

Parameter	Values (eV)
HOMO	-6.833
LUMO	-2.736
HOMO–LUMO gap	4.097
Ionization potential (I)	6.833
Electron affinity (A)	2.736
Chemical hardness (η)	2.048
Chemical potential (μ)	−4.785
Electronegativity (χ)	4.784
Electrophilicity index (ω)	0.438

### 3.4 Molecular electrostatic potential (MEP)

**Figure 3:**
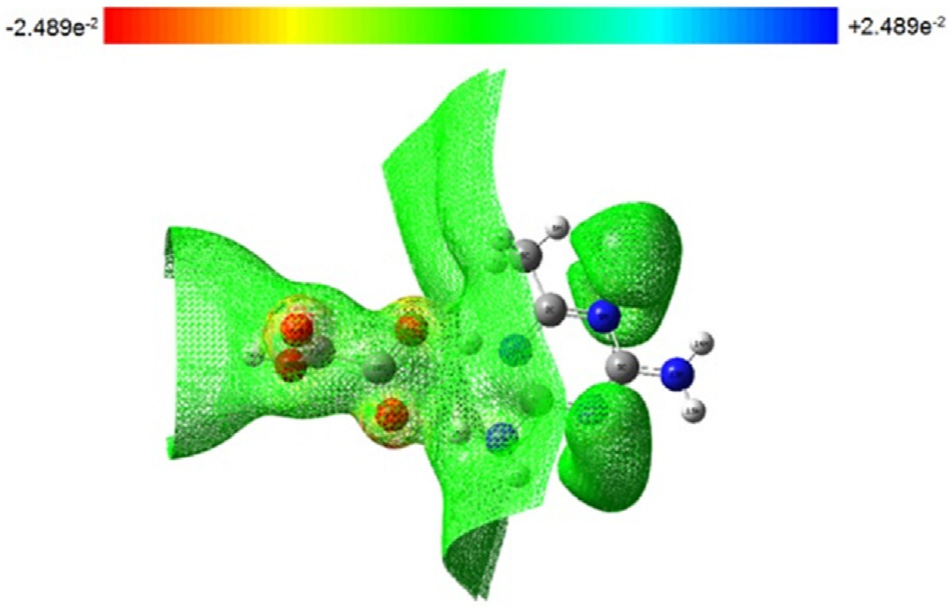
Molecular electrostatic potential map of DMTHO.

**Figure 4:**
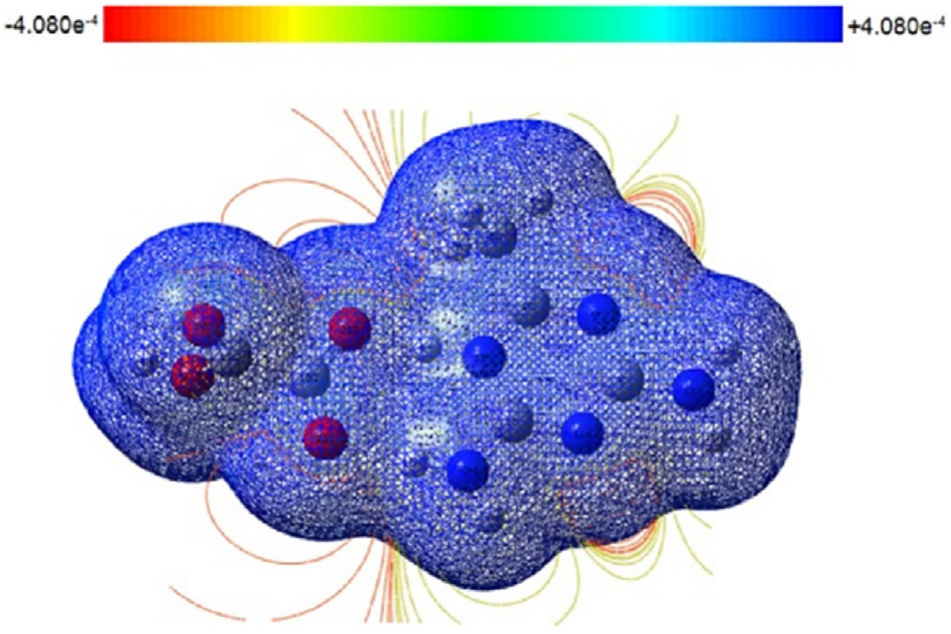
Total density spectrum of DMTHO.

Studies of molecular electrostatic potential are used to comprehend the relationship between a molecule's molecular structure and its properties, including those of pharmaceuticals and biomolecules. Studying molecularly imprinted active sites, recognition, and hydrogen bond production all depend on the MEP, which is connected to electron density. The formation of three-dimensional structure is determined by hydrogen bonding. We can see the variously charged areas of a molecule using ESP maps. The interactions between molecules can be predicted using the charge distributions. Making accurate predictions about how various molecules recognise and bind to one another and then come together to build solid-state structures is crucial in fields like medication design and formulation and will keep this basic science in the forefront. There will be many practical uses for successfully mapping out the structure landscape of these kinds of molecules using structural informatics tools like hydrogen bond propensity and estimated MEP surfaces. In the MEP plot, the electrostatic potential rises from red to orange to yellow to green to blue. Gauss View 4.1 was used to depict the electrostatic potential surface using the B3LYP functional and 6-311G++(d,p) basis set, as illustrated in Figure 3. The ESP ranges from -2.489 × 10^−2^ to +2.489 × 10^−2^ eV. The total density spectrum is shown in Figure 4. The total density spectrum values range from -4.080 × 10^−4^ to +4.080 × 10^−4^ eV. The negative (red) regions were localized on the oxygen atoms which were electron-rich locations that indicates the presence of lone pair and π bonds. The electrophilic sites were composed of them. Therefore, the most vulnerable sites for nucleophilic assault are oxygen electrophilic sites [36, 37]. From the MEP, it can be seen that the oxygen atom (O19) has a negative charge (red) covering it with a minimum value of 0.002489 eV and the amine hydrogen atom (N3H24) has a positive charge (blue) covering it with a maximum value of 0.002489 eV. This demonstrates that intermolecular interactions are real [29].

### 3.5 Nonlinear optical effects

A molecule's hyperpolarizability and intramolecular charge transfer are key factors in determining its nonlinear optical characteristics. Due to Kleinman symmetry, the first-order hyperpolarizability and its 27 components of the 3D matrix were reduced to 10 components [38]. Table 3 displays the dipole moment, polarizability, and initial hyperpolarizability. The dipole moment value, which was 8.2830 Debye, was the highest for component μx. As a result, it was discovered that the molecule was optically reactive in the x-direction. The DFT/B3LYP/6–31++ G (d,p) method was used to calculate the DMTHO compound's dipole moment (μi) and polarizability (α), which resulted in values of 8.4079 D and 54.324 × 10^−24^ esu, respectively. The total value of the first order hyperpolarizability (βtot) is in the range 133.264 10^−31^ esu. The biggest value of hyperpolarizability (107.1421 × 10^−31^) is seen in the β_xxx_ direction which suggests that the significant delocalization of the electron cloud is greater in that direction. Urea is a standard material for studying the nonlinear optical properties of compounds with threshold values of α and β of 3.8312 × 10^−24^ esu and 3.7289 × 10^−31^ esu, respectively [19, 20]. It is found from the computations that the studied crystal is 35.738 times that of urea. Therefore, it can be a challenging material for nonlinear optical applications. Thus, the second-harmonic generation features of the material at the molecular level are caused by the nonzero value of (μi) and the large value of (β) [39, 40].

**Table 3:** Calculated electric dipole moment, polarizability and first order hyperpolarizability of DMTHO.

Parameters	B3LYP/6–311++G (d,p)	Parameters	B3LYP/6–311++G (d,p)
μ_x_	8.2830	β_xxx_	107.1421
μ_y_	0.3181	β_xxy_	-7.9563
μ_z_	-1.4085	β_xyy_	10.2113
μ(D)	8.4079	β_yyy_	-8.8863
α_xx_	-70.9620	β_zxx_	-49.1297
α_xy_	-5.2578	β_xyz_	5.7058
α_yy_	-81.3899	β_zyy_	-0.3335
α_xz_	8.6227	β_xzz_	4.5771
α_yz_	-1.1387	β_yzz_	0.9247
α_zz_	-10.6201	β_zzz_	-2.2403
α_tot_ (e.s.u)	54.324 × 10^−24^	β_tot_ (e.s.u)	133.264 × 10^−31^

### 3.6 Hirshfeld surface studies

**Figure 5:**
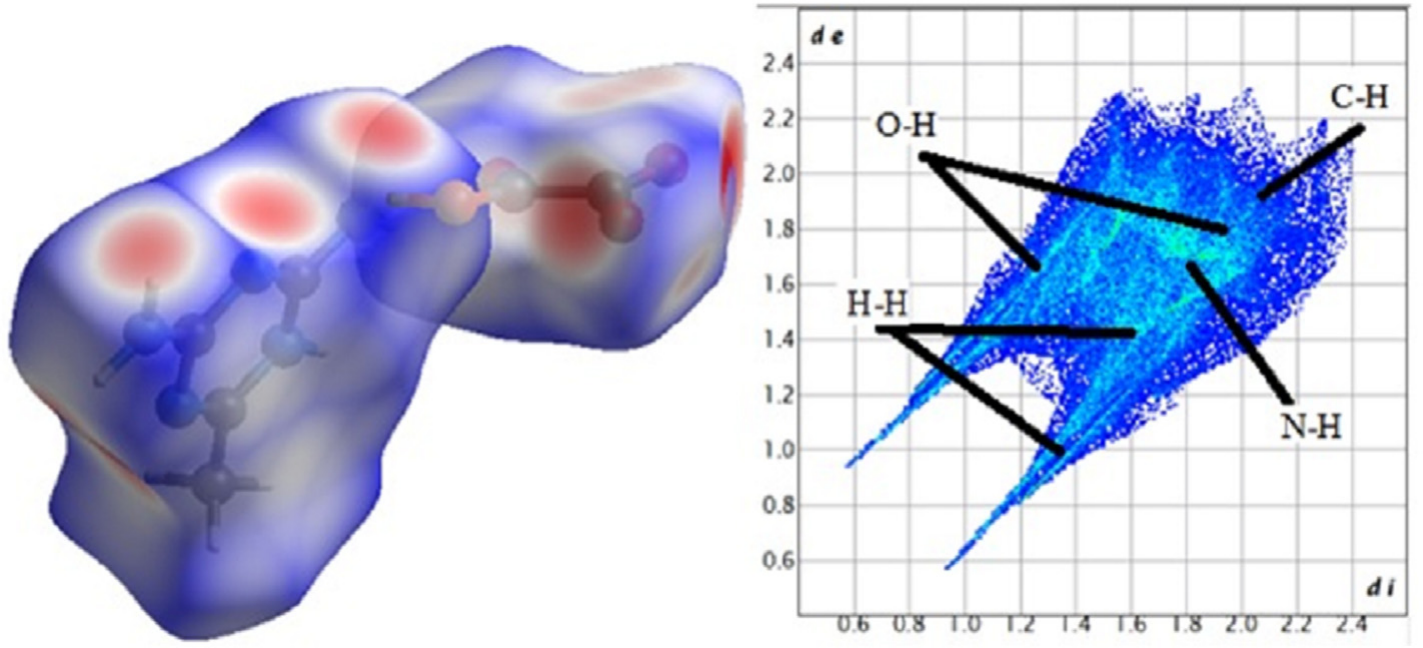
100% fingerprint of DMTHO.

**Figure 6:**
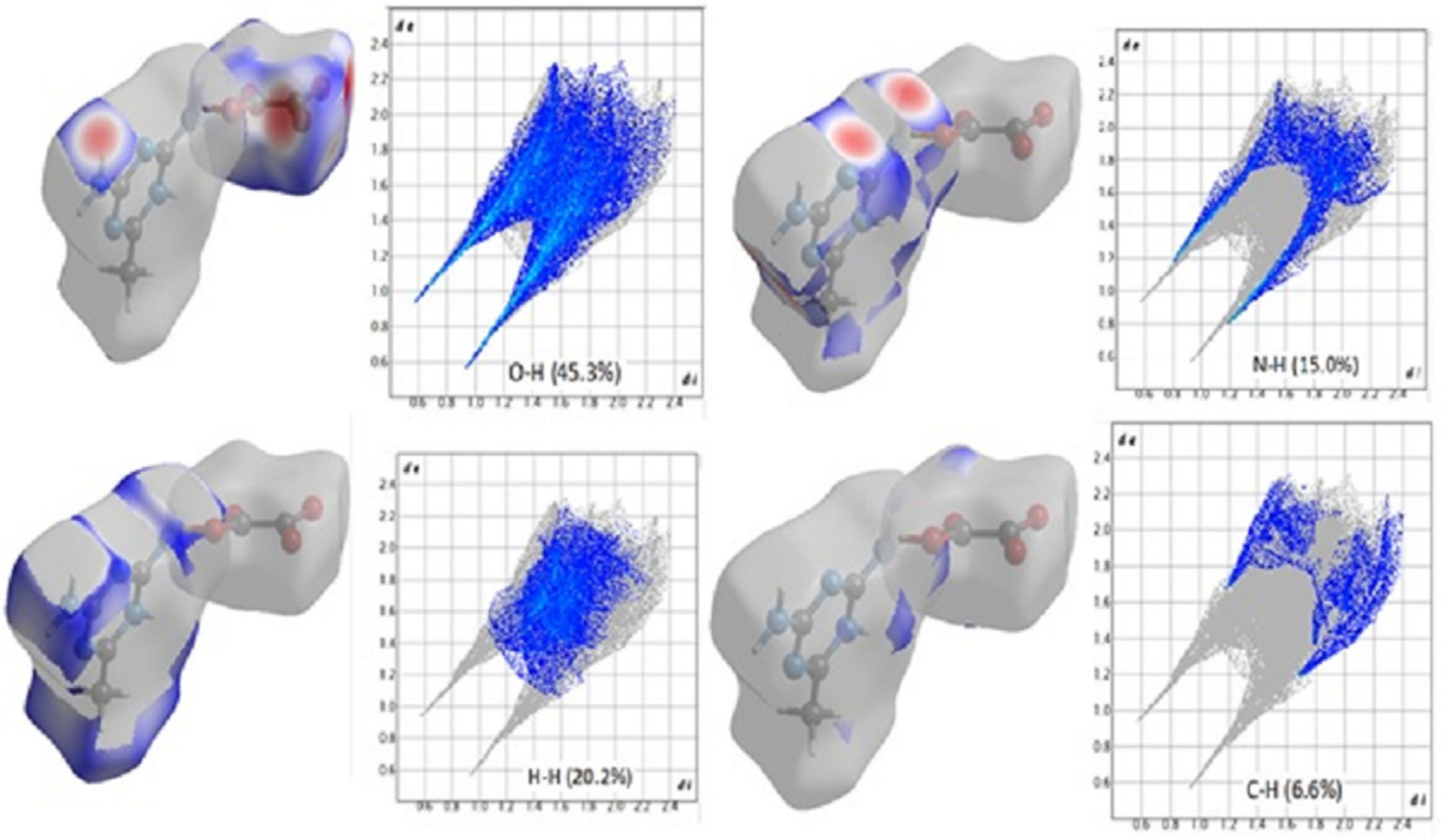
2D fingerprint plots of the studied compound resolved into all contacts [O⋯H/H⋯O; H⋯H; N⋯H and H⋯C].

**Figure 7:**
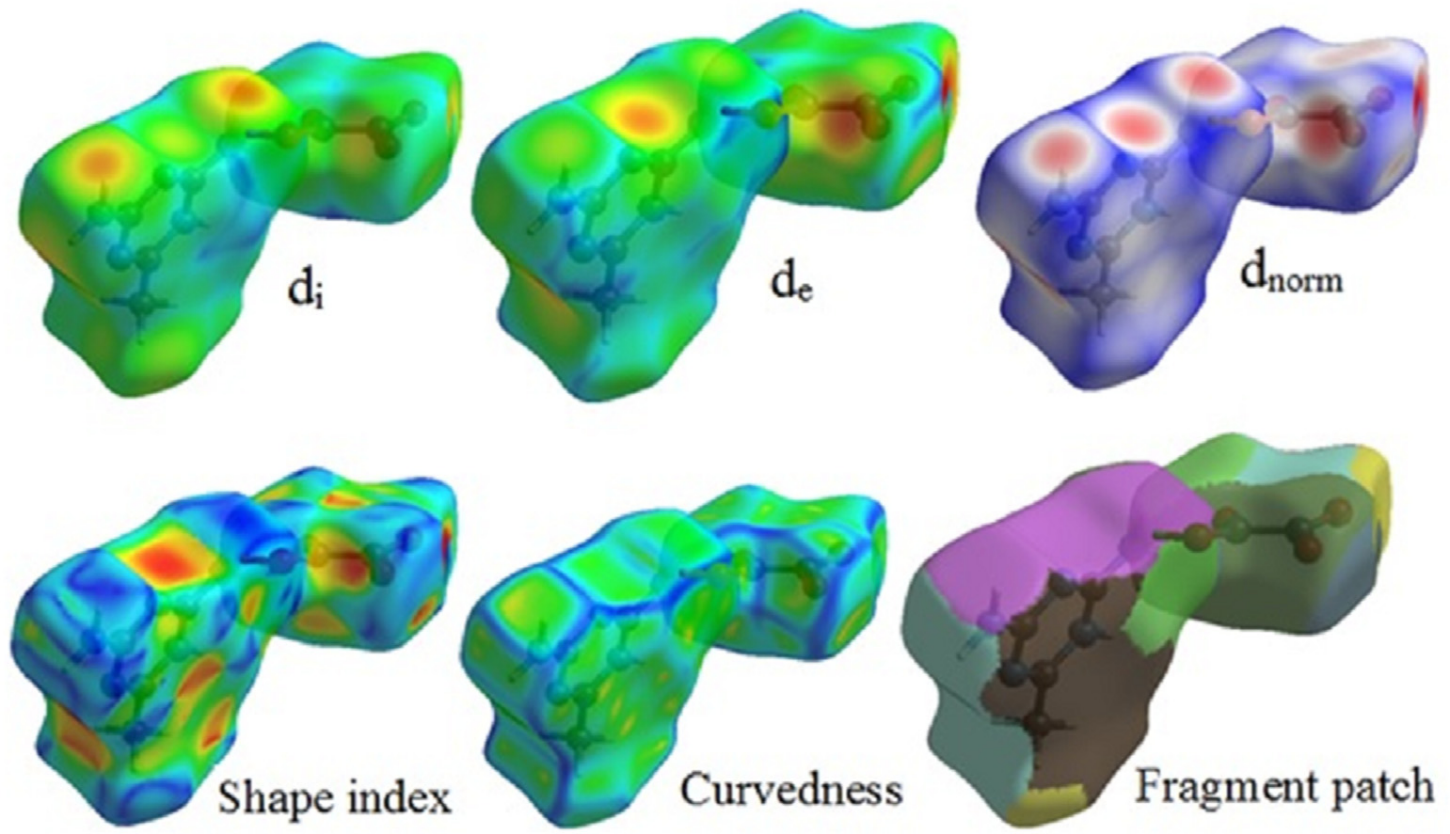
Surface properties (d_i_, d_e_, d_norm_, shape index, curvedness, and fragment patch) of DMTHO.

Crystal Explorer 3.1 was used to conduct Hirshfeld surface studies to investigate the intermolecular interactions of this molecular crystal. A 2D fingerprint (FP) plot with de and di combinations summarises the intermolecular interactions in the crystal. The normalised contact distance (dnorm), which is a symmetric function of these distances to the surface, is inversely proportional to the van der Waals (vdW) radii of the nuclei inside and outside the Hirshfeld surface (di and de, respectively). Red (shorter than vdW separation), white (equal to vdW separation), and blue (longer than vdW separation) were used as the colours to represent the intermolecular interactions (longer than vdW separation). The entire (100%) fingerprint of the crystallised DMTHO is displayed in Figure 5 Figure 6 displays the relative surface area of the relevant interaction along with the separate contributions of O–H, H–H, N–H, and C–H (right) (left). O–H interactions accounted for 45.3% of the Hirshfeld surface of these molecules, which made up the majority of the contributions [38]. O–H and N–H bond interactions are shown by the deep red regions, whereas C–H and H–H bonds are represented by the deep blue regions. Additionally, the fingerprint plots demonstrated the individual contributions of the intermolecular hydrogen bonds to crystal packing through the H–H (20.2%), N–H (15%), and C–H (6.6%) lines. O–C (4.1%), C–C (1.0%), O–O (0.9%), N–O (3.1%), C–N (2.1%), and N–N (1.7%) were other interactions with minor effects. The two big spikes caused by the O–H interaction were a major factor in the intermolecular interactions. The characteristic N–H contacts (‘wings’’) were identified as a weak N–H⋯π interactions. The C–C contacts representing “π–π stacking interactions” are indicated by the red to blue colour gradient. In the bottom left (donor) and bottom right areas of the fingerprint plot, respectively, there are spikes that signify the O⋯H interactions and the HO interactions. The surface properties shown in Figure 7 d_i_ and d_e_ provide information about the crystal packing around the surface of the molecule. Neighboring red and blue triangular patterns that appear on the shape index surfaces, as well as a relatively big and flat green region on the same side of the molecule on the associated curvedness surfaces, all support the existence of π⋯π contacts. The flat zone on curved surfaces indicates π⋯π stacking with little overlap between adjacent molecules. In order to quantitatively investigate all potential non-covalent interactions that could be causing crystal packing, Hirshfeld surface analysis is used [39, 40].

### 3.7 Molecular docking studies

Recent biological research uses the molecular docking technique to find new drugs and understand the precise sites where proteins and ligands attach. It forecasts the protein-ligand interaction with the lowest total energy and the optimal orientation [41, 42, 43]. The best method for analyzing how a target protein interacts with an inhibiting drug is molecular docking studies. Due to their potential use in a number of fields, such as the production of herbicides and the stabilization of polymer photos, 1,3,5-triazines, also known as s-triazines, have long been explored and continue to attract interest. Because of their anticancer effects, several 1,3,5-Triazines are employed in clinical trials to treat lung, breast, and ovarian cancer [43, 44, 45, 46, 47, 48, 49].

**Figure 8:**
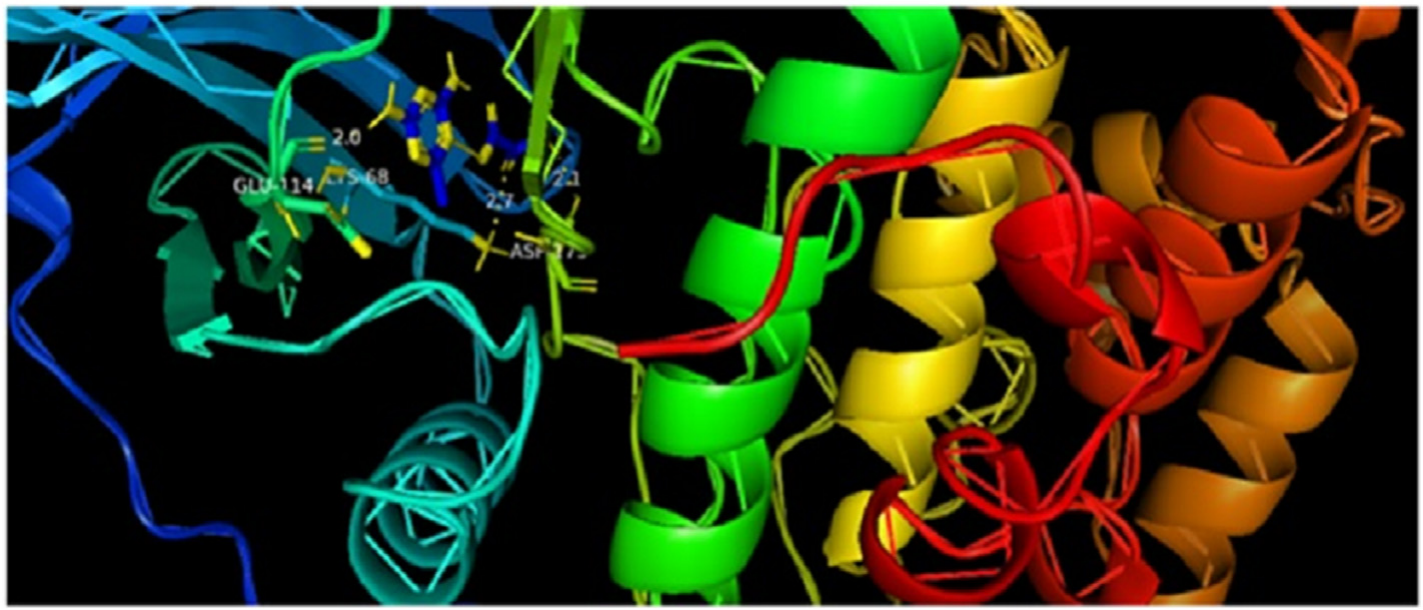
Docking of ligand DMTHO with 6HNY protein.

**Figure 9:**
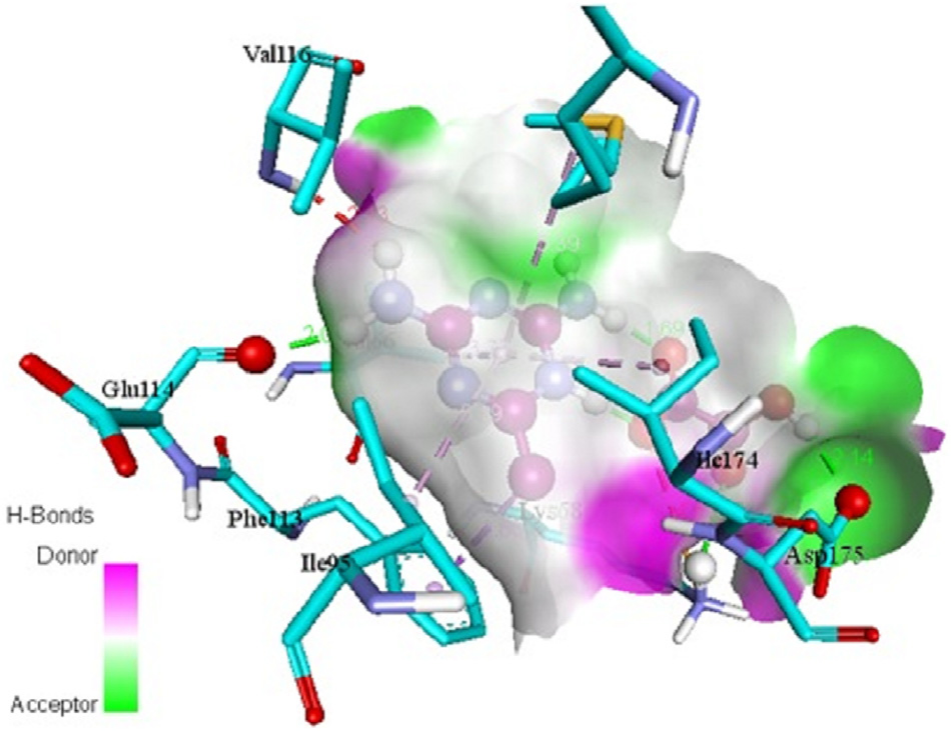
Hydrogen bond interaction of ligand DMTHO with 6HNY protein.

**Figure 10:**
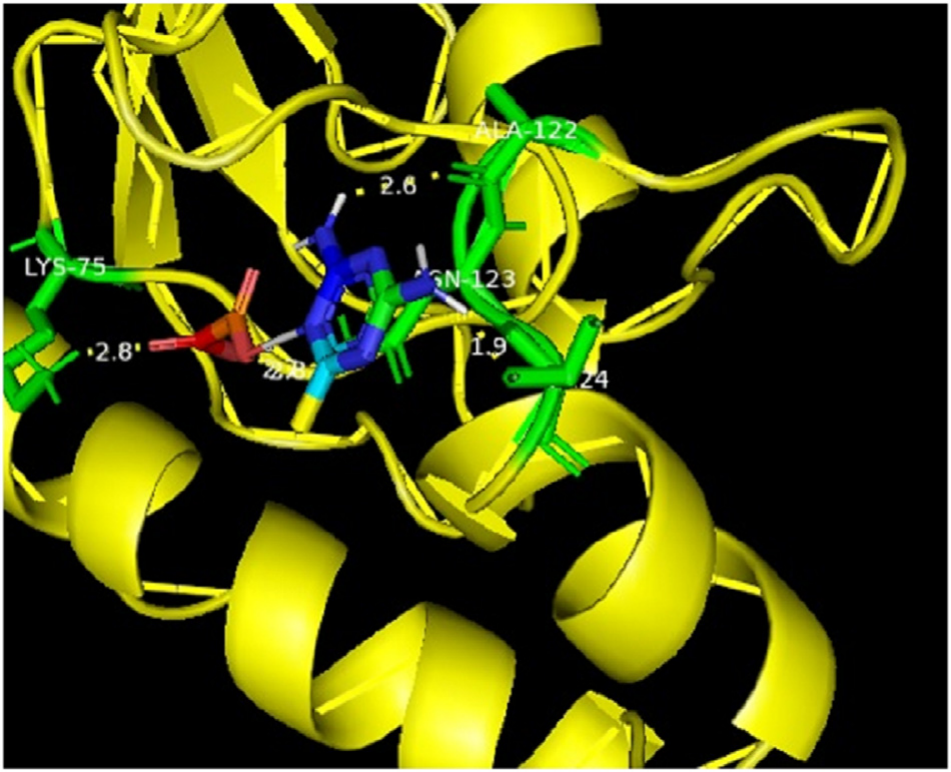
Docking of ligand DMTHO with 2YB6 protein.

**Figure 11:**
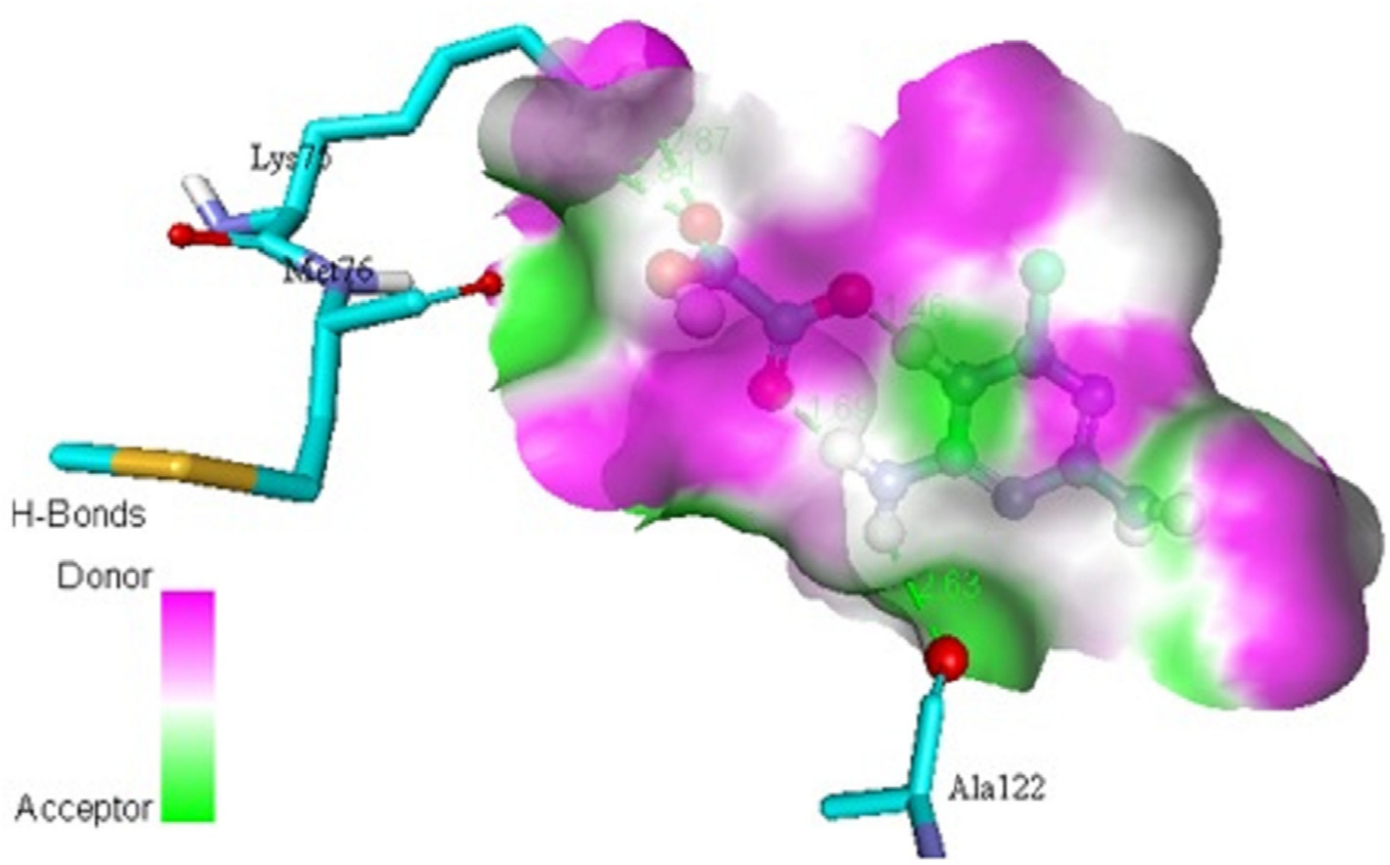
Hydrogen bond interaction of ligand DMTHO with 2YB6 protein.

In addition, the title chemical DMTHO has been employed as a ligand, and the targeted proteins, which were discovered based on the inhibitor, have been linked to ovarian cancer (Rad6 ubiquitin conjugated enzyme [PDB ID: 2YB6] [50, 51]) and breast cancer (protein kinase CK2 [PDB ID: 6HNY]). The minimal energy value is achieved by choosing DMTHO as the proteins' active site. Table 4 lists the residues together with the lowest binding energies for molecular docking (kcal/mol) and bond distance. Figures 8 and 9 show, respectively, the docking and hydrogen bonding interactions of the ligand DMTHO with the 6HNY protein. Additionally, Figures 10 and 11 show, respectively, the docking and hydrogen bonding interactions of the ligand DMTHO with the protein 2YB6. It is acknowledged that the docked ligand DMTHO forms a stable complex with the receptors based on the acquired docking studies. The intermolecular hydrogen bond between the ligand and two distinct proteins is shown as a green dotted line in Figures 9 and 11.

**Table 4:** Molecular docking results of DMTHO molecule with 6HNY and 2YB6 target.

Protein (PDB ID)	Binding Energy (kcal/mol)	No. of Hydrogen Bond	Bonded Residues	Bonding Type	Bond Distance (Å)
**6HNY**	**-7.1**	**3**	LYS68	H-bond	2.73
			GLU114	H-bond	2.00
			ASP175	H-bond	2.14
**2YB6**	**-5.2**	**3**	ALA122	H-bond	2.63
			LYS75	H-bond	2.80
			ASN123	H-bond	2.60

From the molecular docking analysis, it is noticed that DMTHO complex (binding energy value = -5.2 kcal/mol) is stabilized by one hydrogen (acceptor) bond and two hydrogen donor bonds as illustrated in Figures 9 and 11. It also possesses high docking interaction energy of -7.1 kcal/mol. Our compound DMTHO bound with target protein 6HNY through one hydrogen acceptor type interaction and two hydrogen donor type interactions as shown in Table 4. DMTHO can also inhibit the activity of casein kinase (CK2), a known breast cancer-related protein, according to the molecular docking analysis. Molecule docking study result confirms the DMTHO compound has good interactions and properties with the receptors molecules and it is predicted as potential anti-breast cancer drug. As a result, the present study set the basis for further research into developing effective drugs for the treatment of breast cancer.

## 4 Conclusion

Using gradual solvent evaporation, 2,4-diamino 6-methyl 1,3,5-triazinium oxalate single crystals were produced at room temperature. Density functional theory was applied to the 6–311++G (d,p) basis set using the B3LYP functional. NBO analysis and frontier molecular orbital studies revealed an eventual transfer of charge as well as the existence of strong stabilization energy among the molecules. The molecule is biologically active, as evidenced by the modest values of the HOMO-LUMO gap and chemical reactivity. The energy gap of 4.097 eV indicates low chemical reactivity and the occurrence of photochemical phenomena with electron transfer. The MEP map confirms the existence of intermolecular N–H⋯O, N–H⋯N, O–H⋯O, and C– H⋯O interactions and demonstrates the compound's potential for nucleophilic and electrophilic reactions. First-order hyperpolarizability computations reveal that the grown material DMTHO is 35.738 times that of urea. Hyperpolarizability studies acknowledged the presence of NLO activity in DMTHO. Hirshfeld surface analysis, finger plots, and electrostatic potential maps may help to further clarify the percentage of intermolecular interactions and the electrostatic potential distribution of DMTHO. We believe that our DMTHO material candidate will be of use to scientists working on optoelectronics and nonlinear optics in the future. Molecular docking is a structure-based approach that studies and predicts the binding patterns and interaction affinities between ligand and receptor biomolecules. Besides, docking studies establishes the grown material for potential anti breast cancer drug. Computational models' beneficial predictions from docking results could speed up the discovery of anti-breast cancer drugs even more.

## Declarations

### Author contribution statement

K.Ayisha Begam: Conceived and designed the experiments.

N.Kanagathara: Performed the experiments; Wrote the Paper.

M.K.Marchewka: Analyzed and interpreted the data.

An-Ya Lo: Contributed reagents, materials, analysis tools or data.

### Funding statement

This research did not receive any specific grant from funding agencies in the public, commercial, or not-for-profit sectors.

### Data availability statement

Data will be made available on request.

### Declaration of interests statement

The authors declare no conflict of interest.

### Additional information

No additional information is available for this paper.
